# Flower Bats (*Glossophaga soricina*) and Fruit Bats (*Carollia perspicillata*) Rely on Spatial Cues over Shapes and Scents When Relocating Food

**DOI:** 10.1371/journal.pone.0010808

**Published:** 2010-05-25

**Authors:** Gerald G. Carter, John M. Ratcliffe, Bennett G. Galef

**Affiliations:** 1 Department of Biology, University of Maryland, College Park, Maryland, United States of America; 2 Institute of Biology, University of Southern Denmark, Odense, Denmark; 3 Department of Psychology, Neuroscience & Behaviour, McMaster University, Hamilton, Ontario, Canada; University of Bern, Switzerland

## Abstract

**Background:**

Natural selection can shape specific cognitive abilities and the extent to which a given species relies on various cues when learning associations between stimuli and rewards. Because the flower bat *Glossophaga soricina* feeds primarily on nectar, and the locations of nectar-producing flowers remain constant, *G. soricina* might be predisposed to learn to associate food with locations. Indeed, *G. soricina* has been observed to rely far more heavily on spatial cues than on shape cues when relocating food, and to learn poorly when shape alone provides a reliable cue to the presence of food.

**Methodology/Principal Findings:**

Here we determined whether *G. soricina* would learn to use scent cues as indicators of the presence of food when such cues were also available. Nectar-producing plants fed upon by *G. soricina* often produce distinct, intense odors. We therefore expected *G. soricina* to relocate food sources using scent cues, particularly the flower-produced compound, dimethyl disulfide, which is attractive even to *G. soricina* with no previous experience of it. We also compared the learning of associations between cues and food sources by *G. soricina* with that of a related fruit-eating bat, *Carollia perspicillata*. We found that (1) *G. soricina* did not learn to associate scent cues, including dimethyl disulfide, with feeding sites when the previously rewarded spatial cues were also available, and (2) both the fruit-eating *C. perspicillata* and the flower-feeding *G. soricina* were significantly more reliant on spatial cues than associated sensory cues for relocating food.

**Conclusions/Significance:**

These findings, taken together with past results, provide evidence of a powerful, experience-independent predilection of both species to rely on spatial cues when attempting to relocate food.

## Introduction

Differences in foraging behavior might lead to predictable differences in how animals learn about where food is to be found. In particular, animal species can differ in the relative importance that individuals place on spatial versus sensory cues [e.g. 1–6]. For example, there is evidence that seed-caching birds are more likely than non-caching birds to use spatial cues rather than sensory cues, such as color or pattern, to relocate food [7]–[9]. An enhanced reliance on spatial cues for relocating food items might be expected not only in seed-caching species [e.g. 5–6], but also in species that exploit stationary concentrations of food such as flowers. For instance, excellent spatial learning is demonstrated by many nectar-feeding animals (e.g. bumblebees [10] and hummingbirds [11]).

The neotropical bat, *Glossophaga soricina* (Chiroptera: Phyllostomidae) feeds largely on floral nectar, and individuals will revisit the same flower as many as 30 times in a single night [12]. *G. soricina* has an excellent spatial memory, relies heavily on spatial cues and tends to ignore shape cues when relocating sources of nectar [13]–[15]. Even when spatial cues to the location of food become unreliable, *G. soricina* has great difficulty in learning to associate shape cues with food [14], [15]. In Stich and Winter's study [15], an automated, two-arm feeding apparatus alternated the side of an enclosure on which food was available while differences in the shape of the two feeders consistently indicated where food was to be found. Experimentally naïve, captive *G. soricina* required more than 5000 trials before reaching a criterion of 85 percent correct responses to the rewarded shape.

Many neotropical flowers that are pollinated by bats have distinctive scents that are attractive to their pollinators. Many of these scents are sulfur compounds, particularly dimethyl disulfide, which is significantly more attractive to *G. soricina* and its congener *G. commissarisi* than are other floral scent compounds [16]. Because over evolutionary time, floral scents in general, and dimethyl disulfide in particular, have signaled the presence of food to nectar-feeding bats, we suspected that these bats might more readily associate scents than shapes with food and use such scent cues to relocate food sources.

In Experiment 1, we examined the reliance of *G. soricina* on scents, predicting that, unlike shape cues, scent cues would be used to relocate foods. However, a finding that nectar-feeding bats would fail to form associations between scents and food rewards might result from our having used very salient shape cues and relatively weak scent cues. Therefore, in Experiment 2, we repeated Experiment 1 but used weaker shape cues and more salient scent cues.

The scent cue that we used as the rewarded stimulus in Experiment 2, dimethyl disulfide, is a major component of many floral scents, and is strongly attractive to *G. soricina* the first time that they encounter it. Captive-bred, exposure naïve *G. soricina* are significantly more likely to approach test tubes filled with a dilute solution of dimethyl disulfide than test tubes containing other compounds extracted from bat-pollinated flowers [16]. We therefore anticipated that subjects in the present experiment would be even more likely to use the scent of dimethyl disulfide to relocate food rewards than subjects in Experiment 1 that might not use the scent of oregano for that purpose.

Stich and Winter [15] have proposed that when relocating a food source nectar-feeding bats might be more reliant on spatial memory than related fruit-eating bats. They suggest that, although fruiting plants provide resources for some time, a single fruit is collected only once, and thus spatial cues should play a smaller role in relocation of food in fruit-eating than in nectar-feeding bats, such as *G. soricina*, that return many times to feed in precisely the same location.

In Experiment 3, we therefore examined the hypothesis that fruit-eating bats might be less disposed than nectar-feeding bats to rely on spatial cues when seeking to return to a previously profitable food source. Stich and Winter [15] have proposed a continuum among species of neotropical leaf-nosed bats (Phyllostomidae) in reliance on spatial cues when seeking food. Nectar-feeding species that exploit stationary food sources were predicted to be most dependent on spatial cues, insectivorous species to be least dependent on spatial cues, and fruit-eating bats to occupy an intermediate position. Here, we examined reliance on spatial cues when rediscovering food in a fruit-eating phyllostomid, the short-tailed fruit bat *C. perspicillata*. This species is sympatric with *G. soricina* and often roosts with *G. soricina* in the wild; both species forage at ground level in rainforest and share much of their foraging space [17]. One notable difference between the two species is that *G. soricina* has obvious morphological adaptations to nectar feeding [18]–[19], while *C. perspicillata* is primarily a fruit-eating generalist with a considerably broader diet than *G. soricina* [17], [20], feeds on nectar only opportunistically, and lacks dramatic morphological adaptations for exploiting nectar [18]. Consistent with Stich and Winter's hypothesis [15], we expected *C. perspicillata* to show less reliance on spatial cues and more reliance on shape and scent when relocating food than the nectar-feeding *G. soricina* that participated in Experiment 1.

## Methods

### Ethics statement

All experimental procedures in this paper were approved by the Biodome and McMaster University's Animal Care Committee and were carried out in accord with the guidelines of the Canadian Council for Animal Care.

### Experiment 1

#### Subjects

Sixty captive, male *Glossophaga soricina* served as subjects and were housed in the Biodôme de Montréal and maintained on a 12/12 h dark/light schedule in three adjacent rooms (a “test room,” a “waiting room” and a “colony room”) each roughly 3 m^2^×2.5 m high, with a temperature of 25–28°C and 80–100 percent relative humidity. Subjects were maintained on a diet of Nektar-Plus hummingbird food (Nekton Produkte, Pforzheim, Germany), cantaloupe, and a mixture of chopped banana, apple, fig, papaya, and marmoset chow, and had *ad libitum* access to water.

#### Apparatus

**Figure 1:**
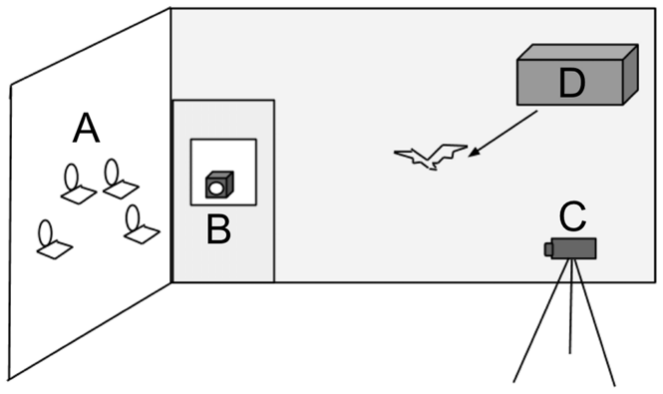
Test Room. Schematic shows A) experimental feeders, B) first video camcorder, C) second video camcorder, and D) bat roost box.

**Figure 2:**
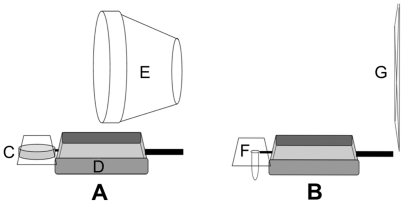
Experimental Feeders. Schematic of feeders used in A) Experiments 1 and 3, and B) Experiment 2, show C) weak scent cue: mesh-covered dish holding herbs or spice, D) metal food dish, E) strong shape cue: flower pot, F) strong scent cue: mesh-covered test tube holding strong liquid scent, G) weak shape cue: flat pattern of pipe cleaners on cage wall.

We tested all bats in the “test room” (Figure 1) that contained an array of feeders (Figure 2). We held extra bats prior to testing in the “waiting room”, which contained a replica of the array of feeders in the test room. The “colony room” housed bats after we had tested them. Food was presented to subjects in feeders (Figure 2A), each consisting of a metal dish, with a tapered terracotta flower pot suspended above it in a unique orientation (shape cue), with the mouth of the pot facing either downwards, outwards/towards the subject, inwards/hidden from subject, or upwards, and a small aluminum-foil dish holding one tablespoon of an herb or spice (scent cue), either rosemary, oregano, cumin, or ginger suspended in front of the food dish and covered by a flap of plastic mesh (Figure 2A). To access the food dish, bats had to fly over the scent cue and in front of the shape cue.

#### Procedure

Following Brodbeck [8] and Thiele and Winter [14], we first trained subjects to visit a food-rewarded feeder in the presence of three other unrewarded feeders. Each of the four feeders had a distinct combination of location, shape cue, and scent cue. Individual bats were then tested with the same four feeders, with one of the three cues (location, scent, shape) removed and the two remaining cues providing conflicting information as to the whereabouts of food. For example, during testing, we presented subjects with one feeder in the previously rewarded location, another with the previously rewarded shape cue, and two control feeders with previously unrewarded shape cues at previously unrewarded locations.

#### Training

Over 10 days, we trained all bats in the test room to feed from only one of four feeders with a distinctive and consistent location, scent (oregano), and shape (outward facing pot). We chose feeder locations by randomly selecting coordinates on the wire grid of the cage. To avoid possible bias towards feeders on the outside of the array (that might have been more accessible to a bat in flight than more centrally located feeders), we flipped a coin to determine which of the two more centrally located feeders would be rewarded. The rewarded feeder contained a mixture of chopped banana, apple, fig, papaya, and marmoset chow. The other three feeders contained the same ingredients as the rewarded feeder mixed with 0.1% w/w quinine, an odorless substance that *G. soricina* finds highly aversive (unpublished observations). This rendered those three feeders non-rewarding whilst controlling for any olfactory or visual cues associated with the food itself.

#### Training tests

Immediately after training, we removed bats to the waiting room so that we could test each subject individually to determine if they were properly conditioned to the reward feeder. During a training test, we presented a subject with the same four feeders in the test room as during training, except that each of the four feeders now contained a piece of banana. Since all feeders contained equal rewards and subjects were tested alone, subjects could not possibly choose feeders based on the presence of quinine, differences in the amount of food in feeders, or the presence or actions of other bats. An experimenter in the adjacent colony room observed the subject's behavior through a Plexiglas window using an infrared sensitive video camera (Nightshot, Sony Corp., NY, USA) and two sources of infrared illumination (HVL-IRM, Sony Corp., NY, USA and IRLamp6, Bat Conservation and Management Inc., Carlisle, PA, USA).

We counted the number of times a subject either landed on a dish or hovered within 15 cm of a dish, facing it, for >3 video frames (0.1 s). If a subject did not choose the reward feeder six times in succession within 20 min, or if it made four incorrect choices in a row, we returned it to the waiting room and tested a new subject. Once a subject had made six consecutive choices of the rewarded feeder, and thus demonstrated that it had learned to go there directly, we immediately gave it a cue test.

#### Cue tests

During a cue test, all feeders were unrewarded, containing only two pieces of cylindrical foam (2.5 cm long, 1.3 cm in diameter). We designed cue tests to investigate subjects' responses to conflicting cues: (1) spatial versus shape cues, (2) spatial versus scent cues, or (3) shape versus scent cues. Each cue test lasted at least 5 min and each ended when the subject made 10 choices, or after 30 min without a subject making 10 choices, whichever occurred first. We observed all cue tests using two infrared-illuminated Sony Nightshot camcorders, one filming straight on and the other at 90 degrees (Figure 1), to resolve any ambiguous observations. We tested ten bats in each of the three conditions described below.

*Location vs. shape*. In location versus shape cue tests, we removed scent cues and, for each bat, switched the shape that had been associated with the rewarded feeder during training with that previously associated with an unrewarded feeder, alternating with which shape we switched the previously awarded shape for each of 10 trials. Thus, each bat chose between a feeder in the previously rewarded location but with a previously unrewarded shape, a feeder associated with the previously rewarded shape but in a previously unrewarded location, and two other feeders that served as controls with previously unrewarded shapes in previously unrewarded locations.

*Location vs. scent*. In location versus scent cue tests, we removed shape cues and, for each bat, switched the scent that had been associated during training with the feeder in the rewarded location with a scent cue that, during training, had been associated with an unrewarded feeder. Thus, bats chose between a feeder scented with a previously unrewarded scent in the previously rewarded location, another feeder with the previously rewarded scent in a previously unrewarded location, and two control feeders in previously unrewarded locations with previously unrewarded scents.

*Shape vs. scent*. In shape versus scent cue tests, we: (1) completely removed the feeder from the location that had been rewarded during training, (2) switched the shapes previously associated with the rewarded feeder with that of a second feeder in a location unrewarded during training and (3) switched the scents previously associated with the rewarded feeder with that of a third feeder in a previously unrewarded location. Bats thus chose between three feeders in previously unrewarded locations: one feeder with the shape it had experienced during training in association with the rewarded feeder, a second feeder with the scent it had experienced during training in association with the rewarded feeder, and a control feeder that had the same unrewarded scent and shape cues that it had experienced during training.

### Experiment 2

#### Subjects

Thirty additional male *G. soricina,* from the same source as those that participated in Experiment 1, participated in the Experiment 2.

#### Apparatus

The apparatus was that used in Experiment 1. However, we chose new feeder locations using the same method as Experiment 1 and used weak echo-acoustic shapes (relatively flat patterns made from pipe cleaners pressed against the cage wall) and four strong scent cues: (1) 1 mL of almond food flavoring (Loblaw Companies, Ltd, Brampton, ON, Canada), (2) 200 µL dimethyl disulfide (VWR International, LLC, West Chester, PA, USA) in 800 µL of water, (3) 1 mL black pepper essential oil (Lotus Brands, Inc, Twin Lakes, WI, USA), and (4) 1 mL of orange food flavoring (Loblaw Companies, Ltd, Brampton, ON, Canada). We placed these liquids in test tubes with their openings covered with fine nylon mesh (Figure 2B). In a pilot experiment, we found that naïve bats from our captive colony, like those tested by von Helversen and others [16], showed a strong preference for test tubes scented with dimethyl disulfide at the concentration that we used in the experiment.

#### Procedure

The procedure was identical to that used in Experiment 1.

### Experiment 3

#### Subjects

Thirty adult *C. perspicillata,* maintained in the Biodôme de Montréal under the same conditions as the *G. soricina* that participated in Experiments 1 and 2, participated in Experiment 3.

#### Apparatus

The experimental situation was the same as that used in Experiment 1 except new feeder locations were chosen.

#### Procedure

The procedure was the same as that used in Experiment 1.

#### Data Analysis

We used Wilcoxon signed rank tests to determine whether the mean percentage of choices towards the two previously rewarded cues were significantly different between Experiments 1 and 3. To maintain an overall alpha of 0.05, we used an alpha of 0.008 for each of the three comparisons of choice distribution that we carried out [24].

## Results

### Experiment 1

#### Training tests

Most subjects rapidly reached the criterion of six correct responses in succession (20/30), while five of the remaining subjects required only a single retest to reach criterion, and all had done so by the fourth retest.

#### Cue tests

**Figure 3:**
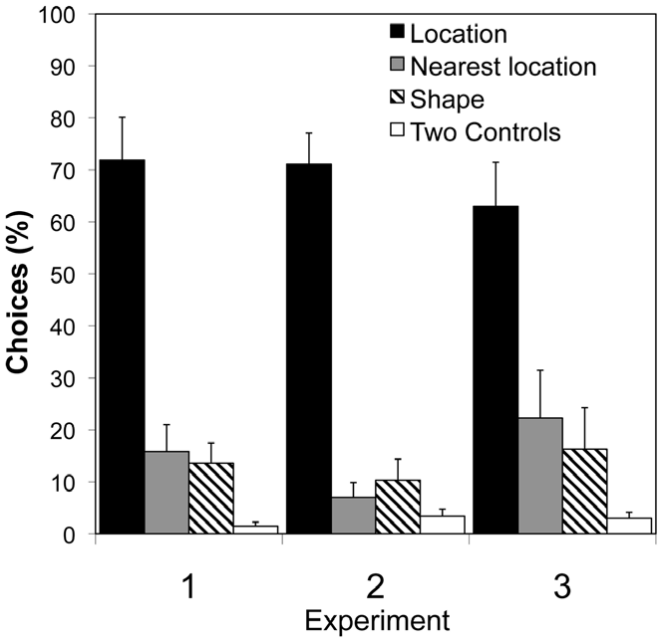
Location versus Shape Tests. Mean percentage of choices (+/− S. E.) of 10 bats are shown for Experiments 1 (flower bats and strong shapes), 2 (flower bats and strong scents), and 3 (fruit bats and strong shapes). Percents do not add up to 100 because previously rewarded shapes or controls can also be nearest locations.

**Figure 4:**
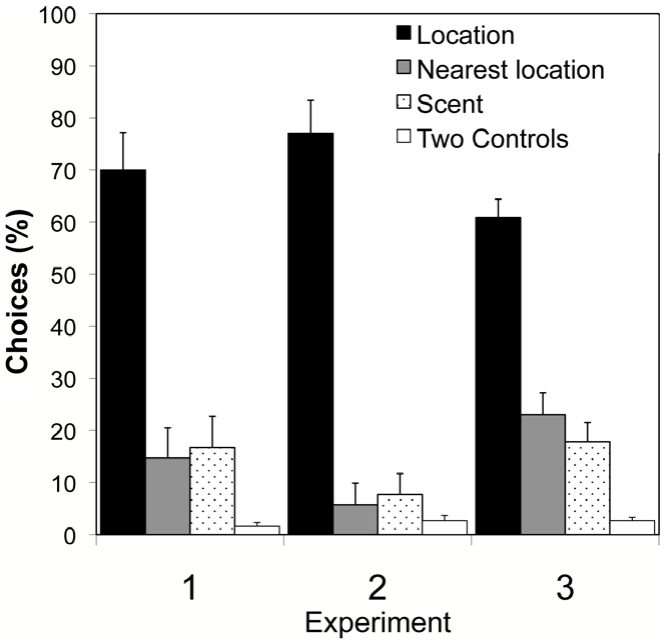
Location versus Scent Tests. Mean percentage of choices (+/− S. E.) of 10 bats are shown for Experiments 1 (flower bats and strong shapes), 2 (flower bats and strong scents), and 3 (fruit bats and strong shapes). Percents do not add up to 100 because previously rewarded scents or controls can also be nearest locations.

**Figure 5:**
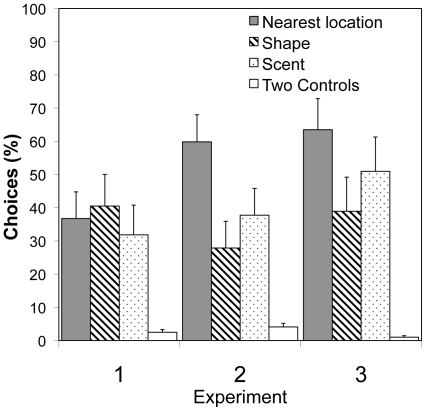
Shape versus Scent Tests. Mean percentage of choices (+/− S. E.) of 10 bats are shown for Experiments 1 (flower bats and strong shapes), 2 (flower bats and strong scents), and 3 (fruit bats and strong shapes). Percents do not add up to 100 because previously rewarded shapes, scents, or controls can also be nearest locations.

Subjects relied heavily on spatial cues when attempting to relocate food. When choosing between location and shape, or location and scent, 19 of 20 bats chose the feeder in the previously rewarded location first. Subjects in these two cue-test conditions returned to that location on approximately 70% of their subsequent choices (Figures 3 and 4), significantly more frequently than they returned to shape (Wilcoxon sign-rank test: *n* = 10, *z* = 21.5, *p*<0.008) or scent (*n* = 10, *z* = 22.5, *p*<0.004). Further, during scent versus shape cue tests, when we had removed the feeder from the previously rewarded position and offered subjects a choice between the previously rewarded scent and shape, they often oriented towards the spot on the cage wall where the rewarded feeder had been located during training. Subjects also chose the feeder nearer the location where the rewarded feeder had been placed at about the same frequency as they visited previously rewarded shapes or scents. During choices between scent and shape, the percentage of their choices did not differ significantly between shape and scent cues (Figure 5).

### Experiment 2

During cue testing in Experiment 2, as in Experiment 1, subjects chose the location rewarded during training far more frequently than they chose either the scent (Wilcoxon sign-rank test: *n* = 10, *z* = 27.5, *p*<0.002) or shape (*n* = 10, *z* = 27.5, *p*<0.002) previously associated with food (Figures 3 and 4). Most surprising, subjects in Experiment 2, when choosing between scent and location, showed no greater tendency to attend to scent cues than had subjects in Experiment 1. Again, as in Experiment 1, in the scent versus shape cue test, subjects in Experiment 2 seemed to remain interested in location, choosing the location closest to that where they had experienced reward during training on more than 60 percent of trials, and attending little to either scent or shape (Figure 5).

### Experiment 3

Like *Glossophaga soricina*, during cue tests of scent versus location and shape versus location, the first choices of *Carollia perspicillata* were highly biased towards location with nine of 10 subjects tested in each condition choosing the previously rewarded location first.

The choices of *C. perspicillata* in Experiment 3 did not differ significantly from the choices of *G. soricina* in Experiment 1 during location versus shape cue tests (*n* = 10, location: *z* = 0.72, *p* = 0.47; shape: *z* = 0.55, *p* = 0.58), location versus scent cue tests (*n* = 10, location: *z* = 0.49, *p* = 0.62; scent: *z* = 0.46, *p* = 0.65), or shape versus scent cue tests (*n* = 10, shape: *z* = 0, *p* = 1; scent: *z* = 1.29, *p* = 0.2).

## Discussion

*Glossophaga soricina* relied heavily on spatial cues when attempting to relocate foods and essentially ignored the associations between a rewarding feeding site and a shape or scent cue in Experiment 1. Our results in Experiment 2 clearly show that relatively low salience of the scent cues used as stimuli in Experiment 1 was not responsible for the lack of reliance of subjects on scent cues when relocating food. Taken together, the results of Experiments 1 and 2 indicate that *G. soricina* is strongly predisposed to rely on cues of location and to ignore both scent and shape cues when attempting to relocate a source of food in situations such as those that we and others [14], [15] have examined.

Possibly, sensory cues such as scents are used primarily at scales larger or smaller than could be studied in our experimental setting. For example, *G. soricina* may use spatial memory to reach known flower locations, then use shape and scent to find flower openings. Similarly, female Mexican free-tailed bats (*Tadarida brasiliensis*, Molossidae) seem to use a step-wise strategy when relocating their own pups amongst what can be millions of others. Spatial memory appears to be used first to locate the general area where a pup was left and olfactory and vocal cues are then used to identify an individual pup in the relevant area [21]–[23].

In all three shape versus scent tests, bats attended to nearest locations as much or more than rewarded sensory cues (Figure 5). It is thus likely that bats were still choosing feeders based on proximity to original location rather than scent or shape. Since both species relied primarily on spatial cues to relocate food, our results were unable to find any difference in use of sensory cues between the flower-feeding *G. soricina* and fruit-eating *C. perspicillata*. Further tests with additional species might determine the extent to which niche-specific strategies for associating particular cues with food rewards exist in bats. For example, Siemers [25] reported evidence that the insectivorous bat *Myotis nattereri* (Chiroptera: Vespertilionidae) can easily learn to ignore location and associate shapes with food.

Theories of associative learning generally share the assumption that stimuli compete for control of behavior [e.g. 26]. Overshadowing [27] is one example of such competition. If two or more stimuli are simultaneously paired with a rewarding event, as occurred in the present experiments, it is often found that response to any one of them will be less than if that stimulus had been the only one paired with reward. Additional evidence of competition between stimuli for control of behavior can be found in studies of blocking [e.g. 28,29] in which the effects of overshadowing are enhanced by training with one stimulus before it is used as an element in a compound stimulus paired with reward. Such effects have been demonstrated in a wide range of both situations and species- fish [30], birds [31], as well as mammals [28], [29], and there is every reason to expect to see them in bats.

The results of the present series of experiment, in which we presented bats with compound stimuli and spatial cues appeared to overshadow both scent and shape cues, are understandable in terms of this fundamental learning mechanism. Because we did not train bats on scent cues alone, and could not therefore compare the control of behavior of scent alone with that of scent as part of a compound stimulus, the evidence of overshadowing of scent by location is not conclusive in our results. Still, the present findings are consistent with the notion that an overshadowing of scent and shape cues by spatial cues is a phylogenetically conserved trait in phyllostomid bats.

The divergence of the phyllostomid bats into a wide variety of ecological niches suggests that they may provide an excellent model system for studies of the evolution of specializations in cognition [32]–[35]. It would be of interest to determine whether: (1) as Stich and Winter [15] suggest in phyllostomid bats, overshadowing of scent and shape cues by spatial cues might be less pronounced in insectivorous than in frugivorous or nectarivorous species of phyllostomids, and (2) prior training with scent or shape cues as signals for the presence of food would reduce reliance on spatial cues in nectar-feeding and fruit-eating phyllostomid bats when they attempt to relocate food. Page and Ryan [32] indirectly demonstrate that this is likely the case for the animal-eating phyllostomid, *Trachops cirrhosus*, when localizing frogs using their mating calls.

In making predictions about the outcome of such experiments, it is important to keep in mind that foraging in rain forest understory, as do many phyllostomid bats, might provide strong general selection for attention to location rather than primary sensory cues while navigating through the environment. We found that both nectar-feeding and fruit-eating bats, born (or living at least 18 years) in captivity, exhibit strong reliance on spatial cues when foraging a relatively few times in a simple, small-scale setting. Taken together with Winter and Stich's demonstration of a similar reliance on spatial cues by nectar-feeding bats feeding many thousands of times in a more complex environment [13], these findings provide compelling evidence of a powerful, experience-independent predilection of the phyllostomid bats studied to date to rely on spatial cues when attempting to relocate food.
